# Xenobiotic-Free Medium Guarantees Expansion of Adipose Tissue-Derived Canine Mesenchymal Stem Cells Both in 3D Fibrin-Based Matrices and in 2D Plastic Surface Cultures

**DOI:** 10.3390/cells9122578

**Published:** 2020-12-02

**Authors:** Caterina M. Suelzu, Virna Conti, Youssef Khalidy, Sara Montagna, Gabriele Strusi, Rosanna Di Lecce, Priscilla Berni, Giuseppina Basini, Roberto Ramoni, Stefano Grolli

**Affiliations:** 1 Dipartimento di Scienze Mediche Veterinarie, Università di Parma, Via del Taglio 10, 43126 Parma, Italy; virna.conti@unipr.it (V.C.); youssef.khalidy@unipr.it (Y.K.); sara.montagna@studenti.unipr.it (S.M.); rosanna.dilecce@unipr.it (R.D.L.); priscilla.berni@unipr.it (P.B.); giuseppina.basini@unipr.it (G.B.); roberto.ramoni@unipr.it (R.R.); 2 Norwich Medical School, University of East Anglia, Norwich, Norfolk NR4 7UQ, UK; g.strusi@uea.ac.uk

**Keywords:** adipose tissue-derived mesenchymal stem cells, stromal vascular fraction, platelet rich plasma, platelet concentrates, veterinary regenerative medicine, 3D culture

## Abstract

Mesenchymal stem cells (MSCs) have been recently introduced in veterinary medicine as a potential therapeutic tool for several pathologies. The large-scale in vitro expansion needed to ensure the preparation of a suitable number of MSCs for clinical application usually requires the use of xenogeneic supplements like the fetal bovine serum (FBS). The substitution of FBS with species-specific supplements would improve the safety of implanted cells, reducing the risk of undesired immune responses following cell therapy. We have evaluated the effectiveness of canine adipose tissue-derived stromal vascular fraction (SVF) and MSCs (ADMSCs) expansion in the presence of canine blood-derived supplements. Cells were cultured on traditional plastic surface and inside a 3D environment derived from the jellification of different blood-derived products, i.e., platelet-poor plasma (PPP), platelet-rich plasma (PRP), or platelet lysate (PL). PPP, PRP, and PL can contribute to canine ADMSCs in vitro expansion. Both allogeneic and autologous PPP and PL can replace FBS for ADMSCs culture on a plastic surface, exhibiting either a similar (PPP) or a more effective (PL) stimulus to cell replication. Furthermore, the 3D environment based on homospecific blood-derived products polymerization provides a strong stimulus to ADMSCs replication, producing a higher number of cells in comparison to the plastic surface environment. Allogeneic or autologous blood products behave similarly. The work suggests that canine ADMSCs can be expanded in the absence of xenogeneic supplements, thus increasing the safety of cellular preparations. Furthermore, the 3D fibrin-based matrices could represent a simple, readily available environments for effective in vitro expansion of ADMSCs using allogeneic or autologous blood-products.

## 1. Introduction

Adipose tissue (AT) is a source of multipotent stromal cells [1]. Although bone marrow (BM) has historically been the preferred source for adult mesenchymal stem cells (MSCs) isolation, the easier access and availability of fat tissue has prompted the use of adipose tissue-derived stromal cells in regenerative therapies in both human and veterinary medicine [2,3]. Although some authors underline differences in their biological features, in vitro and in-vivo studies demonstrate a substantial equivalence between the cells derived from the two sources [4,5,6]. Fat tissue stromal stem cells are isolated, expanded in vitro, and administered in a variety of therapeutic applications, using mainly autologous cells to avoid risks related to immunological reactions and the transmission of infectious disease agents [7]. In veterinary medicine, in particular, adipose tissue derived MSCs (ADMSCs) play a growing role in the therapy of several diseases including, for example, tendonitis, osteoarthritis, bone regeneration, and wound healing [8,9]. More recently, they have been suggested for the treatment of organ and systemic diseases [10,11,12,13]. ADMSCs have also been tested in the treatment of degenerative and traumatic diseases affecting the nervous system [14].

Routine methods for in vitro cell preparation include the enzymatic digestion of a tissue sample collected from the patient and the following in vitro expansion, on the plastic surface of culture vessels, of the obtained cell fraction, known as stromal vascular fraction (SVF). The SVF represents a heterogeneous cell population composed of cell elements proper to the stroma of the adipose tissue, such as resident stem cells/precursor cells, macrophages/monocytes, endothelial cells, and hematopoietic cells [15]. The in vitro expansion of SVF cells aims to select and amplify MSCs, characterized by their plastic adhesion, ability to differentiate towards different mesenchymal cell lineages, and specific markers signature [16]. Recently, the application of SVF in regenerative medicine has been evaluated in both human and veterinary medicine [17,18]. SVF is easily prepared and its heterogeneous composition could contribute to tissue healing through immunomodulation, angiogenesis, and anti-inflammatory effects [15]. Even though regulations applied in veterinary medicine are less restrictive in comparison to the Good Manufacturing Practice (GMP) compliance required in the human field, the preparation of MSCs must be carried out respecting strict parameters to guarantee adequate safety for the patient [19,20]. From this point of view, the supplementation of growth medium with fetal bovine serum (FBS) during in vitro amplification processes represents a major safety concern in the preparation of MSCs suitable for clinical application. The use of this growth-promoting xenobiotic component can cause adverse reactions following cell therapy, with potential risks for patients, mainly when cultured cells are administered by systemic route (for example, by intravenous injection) [19]. Another issue of in vitro expansion of MSCs is associated with the time period needed for the preparation of an adequate number of cells since the collection of the adipose tissue. An average time of 2–3 weeks is routinely requested, but an even more extended time is necessary when systemic administration is applied. This latter approach requires a high number of cells and, eventually, repeated treatments. Despite a limited number of published works and the differences in the therapeutic protocols described, for the systemic administration of MSCs in dog and cat, a cell dosage of 0.5–2 × 10^6^ cells/kg is required [21,22]. Consequently, the production of an adequate number of cells in a short time could be highly demanding and hard to satisfy, especially for large-size patients, preventing an adequate therapeutic approach.

A possible solution to safety concerns related to the use of FBS as a medium supplement may be its substitution by species-specific blood-derived supplements, such as serum, platelet-rich plasma (PRP), or platelet lysate (PL). This approach has been considered in the recent years for the preparation of human MSCs, where the restrictive regulations led either to the formulation of serum-free culture media or to the substitution of FBS by products of human origin, mainly PL [23]. The literature in veterinary medicine, indeed, does not provide much information about the effectiveness of platelet derivatives on MSCs culture and the chance to replace FBS with non-xenobiotic products [24,25,26]. Furthermore, no canine or feline species-specific serum substitutes are commercially available. The recent scientific literature underlines that the use of species-specific serum, and/or platelet derivatives, could fulfil a further critical requirement in MSCs culture, providing a three-dimensional (3D) scaffold enriched by growth factors, which mimic a more complex and physiologic environment in comparison to the canonical plastic surface of culture vessels [27,28].

The present work aims to evaluate the possibility of expanding dog ADMSCs by introducing new approaches to improve the timing of cell preparation and to increase the safety of their clinical application. Allogeneic and autologous blood-derived products were evaluated as substitutes for FBS in cell expansion. In particular, serum, PRP, and PL were used. It was also assessed whether the three-dimensional matrix prepared by the gelation of different blood-derived products can increase cell proliferation compared to the traditional plastic surface. Even in this case, the possibility of using homospecific supplements to replace the FBS was considered. The 3D fibrin-based matrix was also used to evaluate the possibility of expanding the SVF in this environment. The use of a xenobiotic-free matrix capable of accelerating the in vitro expansion of cells intended for the clinical application would improve the timing of application and the safety of regenerative medicine for dogs.

## 2. Materials and Methods

### 2.1. Reagents

Media, supplements, and other cell culture reagents used for cell culture were from Gibco, unless differently specified. The plastic labware was from Jet Biofil, Carlo Erba, Italy.

### 2.2. Specimens Collection

Canine subcutaneous or abdominal adipose tissue samples (2–5 g) were collected from healthy donor animals at the local Veterinary Teaching Hospital, during ovariohysterectomy or other elective surgeries. The patients underwent routine clinical, biochemical, and hematological examination prior to the surgical procedure. The owners of the animals gave an informed consensus.

### 2.3. Preparation of ADMSCs by Enzymatic Digestion of Adipose Tissue

After the samples collection, adipose tissue fragments were stored in complete phosphate-buffered saline solution (cPBS) containing penicillin (50 U/mL), streptomycin (20 µg/mL), and amphotericin B (2.5 µg/mL) before being processed (maximum 2 h). Then, 1–1.5 g of adipose tissue were fragmented in 0.3–0.5 cm^3^ pieces by means of a sterile scalpel under a cell culture hood and were then transferred to a 15 mL conical centrifuge tube. The tube contained a solution of 0.1% *w*/*v* collagenase type I, prepared in DMEM low glucose, supplemented with 50 U/mL penicillin, 20 µg/mL streptomycin, 2.5 µg/mL amphotericin B, at a ratio of 5 mL of medium per gram of minced tissue. The enzymatic digestion was carried out in a water bath at 37 °C, in mild agitation for 60 min. The cell suspension was then filtered through a nylon filter (mesh 100 µm) and centrifuged at 190× *g* per 15 min. The cell pellet was re-suspended in 3 mL of maintenance medium (mDMEM), consisting of DMEM low glucose supplemented with 10% *v*/*v* FBS, penicillin 50 U/mL, streptomycin 20 µg/mL, amphotericin B 2.5 µg/mL, and then seeded in 25 cm² culture flasks. The cells were maintained in an incubator at 37 °C in 5 % CO₂ atmosphere, renewing the medium every 72 h. When the cells reached about 80% of confluence, they were detached using 0.05% Trypsin-EDTA in cPBS. The cells were then expanded until P3-P4 when they were used for the experiments described below. When needed, cells were cryopreserved in liquid nitrogen using a freezing medium consisting of 50% (*v*/*v*) FBS, 10% (*v*/*v*) dimethyl sulfoxide, and 40% mDMEM.

The pellet obtained from the fat tissue digestion described above and resuspended in 0.5–2 mL of mDMEM represents the stromal vascular fraction (SVF). This product was seeded on plastic or alternatively inside a 3D fibrin-based matrix according to the different experimental set-up, as described below.

### 2.4. Platelet-Rich Plasma and Platelet Lysate Preparation

For platelet-rich plasma (PRP) preparation, peripheral venous blood was collected into a 15 mL conical centrifuge tube containing 1.5 mL of sodium citrate 3.8% *w*/*v* anticoagulant solution (10x). PRP was prepared by a double centrifugation method as already described [29]. After the first centrifugation at 180× *g* for 20 min, the erythrocyte fraction was discarded, while the plasma, enriched with platelets, was centrifuged at 900× *g* for 15 min, obtaining a platelet-poor plasma (PPP) and a cell pellet. The platelets were resuspended in a small volume of PPP, counted, and then accordingly resuspended at a final concentration of 0.5–1 × 10⁹ platelets/mL in an adequate volume of PPP, to obtain the PRP. To obtain the platelet lysate (PL), PRP was aliquoted in 2 mL Eppendorf tubes, frozen at −80 °C and then thawed at 37 °C (2 cycles) to lysate the platelets and to release the growth factors contained therein. The lysate was then centrifuged at 13,000× *g* at 4 °C for 15 min to remove the platelet membranes and fragments. PPP, PRP, and PL were used as a substrate for ADMSCs growth, forming a 3D fibrin-based matrix (see Section 2.7).

### 2.5. Preparation of Canine Blood-Derived Supplements Used for ADMSCs Growth

To assess ADMSCs’ growth in the presence of canine venous blood-derived supplements as substitutes for FBS, different preparations were evaluated, i.e., allogeneic and autologous serum prepared from PPP, allogeneic, and autologous PL and PRP. Blood samples were collected in 3.8% sodium citrate anticoagulant solution (10×) as previously described (see Section 2.4). For the preparation of serum, PPP was induced to clot in a 15 mL conical centrifuge tube by adding 10% *v*/*v* calcium gluconate 100 mg/mL. After 2 h, the tube was centrifuged at 1500× *g* for 20 min to separate the serum from the clot. Allogeneic PL and allogeneic canine serum were prepared by mixing PL or serum from three different animals. Autologous serum and PL were prepared from the same animal donors of the cells used in the study.

### 2.6. Thrombin-Enriched Plasma Preparation

To prepare the solution enriched in thrombin used for 3D matrix preparation, 10 mL of PPP were supplemented with 10% (*v*/*v*) calcium gluconate 100 mg/mL (S.A.L.F., Cenate Sotto (Bergamo), Italy), to induce plasma coagulation. After an incubation of at least 2 h at 37 °C, the clot was mechanically broken and then centrifuged at 1500× *g* per 20 min. The obtained solution rich in thrombin was aliquoted in 2 mL tubes and stored at −20 °C until used for 3D matrix preparation.

### 2.7. 3D Fibrin-Based Matrix Preparation

**Figure A1:**
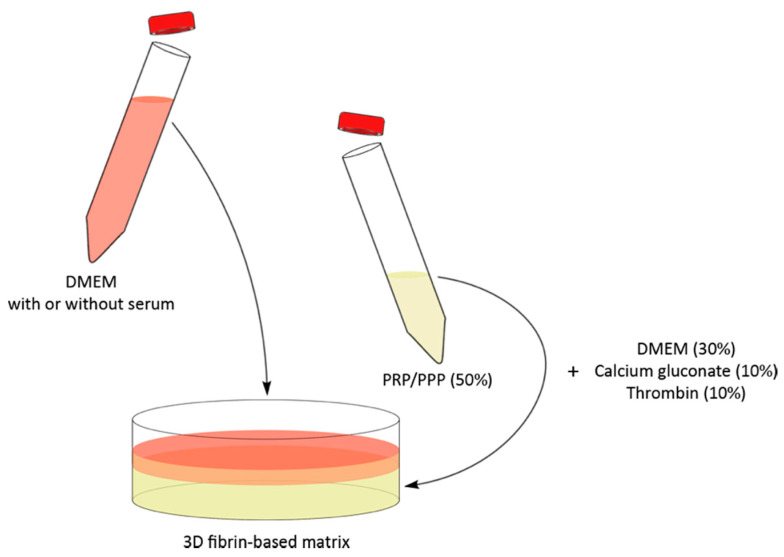
Graphic representation of 3D fibrin-based matrix preparation. The 3D matrix (yellow) was prepared by jellifying platelet concentrates or plasma. The overlying medium could contain or not supplementation (see different experimental set-ups described in Materials and Method). FBS was not used to prepare the 3D matrix. Cells were seeded inside the 3D fibrin-based matrix.

A 3D fibrin-based matrix was obtained combining 30% mDMEM, 50% PPP, PRP, or PL (see Figure A1), 10% thrombin enriched plasma, and 10% (*v*/*v*) calcium gluconate 100 mg/mL (S.A.L.F., Italy). The 3D fibrin-based matrix was then evaluated as an environment capable of supporting the expansion of ADMSCs and SVF. The cells were embedded in the matrix immediately before gelation, initiated by calcium gluconate supplementation. The average time for initial gelation of the 3D matrix was 5–10 min, while 30–60 min were needed for complete gelation. After gelation was completed, the 3D gel was layered with an appropriate amount of culture medium, to sustain the cell growth. The final volume of the 3D fibrin-based matrix was 50 µL when prepared in 96-well plates, or 2 mL when prepared in 35 mm Petri dishes cultures (see below).

### 2.8. Enzymatic Digestion of Fibrin-Based 3D Matrix

To compare ADMSCs growth on the plastic surface with that inside the 3D fibrin-based matrix, a method to recover cells grown within the gel in 35 mm Petri dishes has been developed. For this purpose, a mix of 0.05% (*w*/*v*) collagenase type I and 0.05% (*w*/*v*) dispase dissolved in mDMEM without FBS was used. A fibrin-based matrix was cut into 2–4 mm^3^ pieces by means of a sterile scalpel blade and then transferred into a 15 mL conical tube containing 4 mL of the enzymatic mix. The enzymatic digestion was carried out at 37 °C for 60 min, in mild agitation. The digested sample was then centrifuged at 190× *g* for 15 min. Subsequently, the cell pellet was resuspended in 1–2 mL of DMEM; an aliquot was used for cell count in a Burker chamber, while the remaining cells, according to the experimental set-up, were re-embedded in a 3D fibrin-based gel at a density of 6000 cells/cm^2^ or cryopreserved.

### 2.9. Evaluation of ADMSCs Growth: MTT Assay and Direct Cell Count

To evaluate ADMSCs growth in the presence of different culture medium supplements (i.e., in the presence of FBS or canine blood-derived supplements) and environments (i.e., 2D plastic surface or 3D fibrin-based matrix) two different experimental approaches were used. MTT assay was used in small-scale experiments (performed in 96 well plates), while direct cell count was used to determine the cell number in large scale experiments (performed in 35 mm diameter Petri dishes). The different experimental procedures are described below.

### 2.10. Plastic Surface Versus 3D Fibrin Matrix to Expand ADMSCs

To assess the role of different culture environments on ADMSCs growth rate, the cells were cultured either on the traditional plastic surface (2D culture) or inside a 3D fibrin-based matrix (see Figure A1 and Section 2.7 for its preparation). The cell growth was then evaluated by direct cell count (35 mm diameter Petri dishes) and MTT assay (96 well plates).

**Figure A2:**
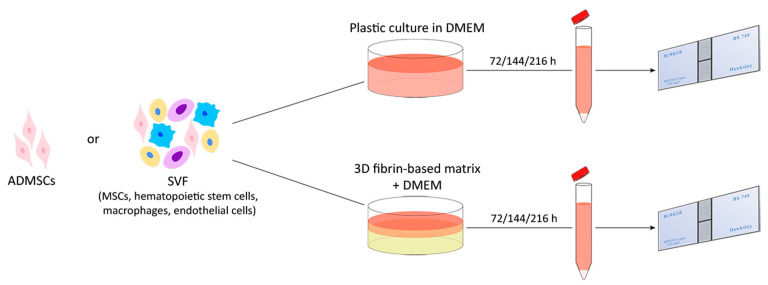
Graphic representation of experiments performed with ADMSCs or SVF to determine cell growth by direct cell count, on plastic surface or inside a 3D environment. The same experimental scheme was applied, while different culture times and supplementations were used, as described in the Material and Methods section. Cells were maintained in 3.5 cm Petri dishes (6 well plates).

Direct cell count: to compare the ADMSCs growth on plastic and inside the 3D matrix by direct cell count, P3-P4 cells were seeded at a density of 6000 cells/cm², in 3.5 cm Petri dishes, both on the plastic surface and inside the 3D matrix prepared from PPP jellification as described above (see also Figure A2). After 48, 72, 144, and 216 h cells grown on the plastic surface were trypsinized, while the matrix was digested as previously described (see Section 2.8). Cells were then counted in a Burker chamber. Cells vitality was assessed by Trypan Blue assay.

**Figure A3:**
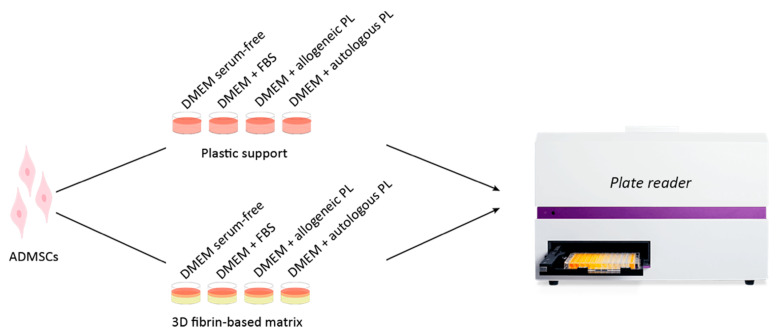
Graphic representation of experiments performed with ADMSCs to determine cell growth by colorimetric MTT assay, on plastic surface or inside a 3D environment. The same experimental scheme was applied, while different culture times and supplementations were used, as described in the Material and Methods section. Cells were maintained in 96 well plates.

MTT assay: the same experimental set-up was repeated in 96-well plates and cell growth was assessed by MTT assay (see Figure A3). For this purpose, 5 × 10^3^ or 10 × 10^3^ cells were seeded in each well and cultured on plastic surface or inside the 3D matrix in a final volume of 150 µL. After 48, 72, or 96 h, 50 µL of medium were removed from each well and 20 µL of a 5 mg/mL solution of MTT in phosphate-buffered saline (PBS), pH 7.4 were added. After 4 h, formazan salts were solubilized adding 100 µL of 10% SDS in 0.01 M HCl. Formazan absorbance was recorded after overnight incubation at 570 nm by a Victor reade spectrophotometer (Perkin Elmer, Groningen, Netherland). Each experiment was repeated five times, and for each experiment 6 replicates were prepared.

### 2.11. PPP Versus PRP for the Expansion of ADMSCs in 3D Fibrin-Based Matrix

To compare ADMSCs growth inside 3D matrices prepared with PRP or canine PPP, 6000 cells/cm² were included in matrices and cultured for 72 or 144 h in 3.5 cm Petri dishes as described above. 3D matrices were then digested, and cells counted in a Burker chamber.

### 2.12. Comparison of the Efficacy of Allogeneic Canine Serum and FBS to Maintain ADMSCs Growth on Plastic Surface and within a 3D Fibrin-Based Matrix

To evaluate the possibility to substitute FBS by canine serum to expand ADMSCs, 5000 cells/well were cultured in 96 well plates in the following conditions: (1) in the presence of 10% FBS supplemented medium (mDMEM); (2) in the presence of 10% allogeneic canine serum-supplemented medium; (3) without any serum supplementation (serum-free medium). The final volume of culture medium in each well was 150 µL. The same set-up was repeated to compare the growth of ADMSCs seeded within a 3D fibrin-derived matrix. In this case, the 3D matrix (50 µL final volume) was prepared as previously described (see Section 2.7) and then different media supplemented with: (1) 10% fetal bovine serum; (2) 10% allogeneic canine serum, or (3) DMEM alone, which was added to a final volume of 150 µL/well. After 72 h, 50 µL of medium were removed from each well and 20 µL of a 5 mg/mL solution of MTT in PBS, pH 7.4, were added. After 4 h, formazan salts were solubilized and quantified as previously described. Each experiment was repeated three times, and for each experiment 6 replicates were prepared.

### 2.13. Evaluation of ADMSCs Growth on Plastic Surface in the Presence of Different Amounts of Allogeneic Canine PL

The impact of different PL concentrations on ADMSCs growth was evaluated by MTT test in 96-well plates. Then, 5000 ADMSCs were seeded on plastic surface in DMEM, in the absence of serum, in the presence of 10% FBS or in the presence of allogeneic canine PL ranging from 5 to 20%. The cells were cultivated for 72 h, and then the MTT assay was performed as previously described. The experiment was repeated three times and six replicates were used for each experiment.

### 2.14. Evaluation of ADMSCs Growth on Plastic Surface and Inside a 3Dfibrin-Based Matrix in the Presence of Autologous Canine Serum

These experiments were set up to verify the possibility to grow ADMSCs using autologous serum as growth supplement, instead of either FBS or allogeneic canine serum. Canine serum was prepared from PPP as described above (see Section 2.5). ADMSCs metabolic activity was evaluated by MTT assay both on cell grown on plastic surface and inside the 3D fibrin matrix, cultivating cells in serum-free medium, in medium containing 10% FBS and in medium supplemented with 10% autologous canine serum. In the experiments performed within the 3D fibrin matrix, the serum used for the preparation of the matrix (Figure A1) was also autologous, i.e., prepared from the same animal donor of ADMSCs. The experiment was repeated three times with different ADMSCs preparations; six replicates were used for each experiment.

### 2.15. SVF Cells Growth in 3D Fibrin-Based Matrix Versus 2D Plastic Surface: Direct Cell Count

After the digestion of the adipose tissue (Section 2.3), the SVF cells were counted and either seeded on plastic flasks or directly embedded in the 3D matrix, in equal aliquots. In this case, due to the heterogeneity of the cell sample, it was not possible to determine a priori the precise number of MSCs present in the stromal vascular fraction. When a confluence of about 90% was achieved on plastic (no longer than 192 h), cells were trypsinized or extracted from the gel by digestion and counted as previously described (Section 2.8).

### 2.16. Determination of Cell-Doubling Time and Cell-Doubling Number

Cell-doubling number (CDn) and cell-doubling time (DT) were calculated as suggested by Vidal et al. [30] and Roth [31]. The two parameters were evaluated as follows:

CD = 𝑙𝑛 (𝑁𝑓/𝑁𝑖)/𝑙𝑛 (2),(1)

DT = 𝐶𝑇/𝐶𝐷n,(2)

where 𝐶𝑇 is the cell culture time, 𝑁𝑓 is the final number of cells, and 𝑁𝑖 is the initial number of cells.

### 2.17. Phenotypic Characterisation of Cell Cultures

The cell cultures were analyzed for the expression of a panel of markers using RT-PCR. ADMSCs grown on plastic or maintained inside the 3D scaffold were analyzed at P3. SVF cells grown directly inside the 3D fibrin-based matrix were characterized after 8 days of in vitro culture. For both cell populations, the analysis was performed on three different cell preparations. Total RNA was extracted from 1.5 × 10^6^ cells using the Nucleospin^®^ RNA II kit (Macherey-Nagel) following the manufacturer’s instructions. cDNA was prepared via reverse transcription of 1.5 μg of total RNA using RevertAid™First Strand cDNA Synthesis Kit (Fermentas). PCR was performed using 2 μL of cDNA solution. The final PCR mixture was composed of 1× amplification buffer with 2.5 mM MgCl_2_, 10 mM dNTP Mix (Thermo Scientific), 0.25 μM specific forward and reverse primers, 1 U Dream Taq (Thermo Scientific) in a final volume of 25 μL. Table A1 and Table A2 report the list of the analyzed genes, their gene accession number, the sequence of forward and reverse specific primers, and the length of the relative amplicons. All PCR experiments were performed using the following protocol: denaturation at 94 °C for 30 s; annealing at 55 °C for 30 s; extension at 72 °C for 30 s, repeated for 35 cycles. Amplicons were separated on agarose gel (1.5% *w*/*v*) in TAE buffer stained with 3.5 μL ethidium bromide (10 mg/mL) and visualized under UV light with a transilluminator. Images were acquired by a Canon digital camera.

### 2.18. ADMSCs Differentiation

To assess the differentiation ability of ADMSCs, cells were cultured either on the traditional plastic surface (2D culture) or inside a 3D fibrin-based matrix and then transferred in 6 well/plates to undergo adipogenic and osteogenic differentiation.

Adipogenic differentiation: ADMSCs derived from 2D and 3D cultures (P3) were seeded in six-well plates at a density of 6 × 10^3^ cells/cm^2^, in complete medium. When the cells reached a confluency of about 80%, they were treated with adipogenic differentiation media (StemPro Adipogenesis Differentiation Kit, Fisher Scientific, Hampton, NH, USA). ADMSCs differentiation was induced following manufacturer instruction. After 21 days the cells were fixed with 70% ethanol and processed for Oil Red O staining [32]. To compare the adipogenic potential of the two different cell populations, 10 micrographs were taken for each of them and 3000 cells were counted. Cell differentiation potency was measured calculating the percentage of Oil Red O positive cells. The count was repeated for three different experiments.

Osteogenic differentiation: ADMSCs were seeded in six-well plates at a density of 6 × 10^3^ cells/ cm^2^, in complete medium. When cells reached a confluence of about 80%, they were treated with osteogenic induction medium (complete medium supplemented with 100 nM dexamethasone, 10 mM glycerophosphate, and 0.250 mM ascorbic acid). The medium was changed every 2–3 days. After 21 days the cells were fixed with 1% paraformaldehyde and processed for alizarin red staining [32].

### 2.19. Statistical Analysis

Data are presented as mean ± SD. Statistical differences were estimated through two-way ANOVA, using the software Prism 8 (GraphPad, San Diego, CA, USA). With the presence of statistically significant differences (*p* < 0.05), mean values were subjected to Tukey’s test for multiple comparisons.

## 3. Results

### 3.1. Direct Cell Count and MTT Assay Can be Used to Evaluate Canine ADMSCs Cell Growth in Different Environments

To evaluate ADMSCs proliferation in different environments, i.e., plastic surface and 3D fibrin-based gel, two approaches were employed: direct cell counts in large scale set-up (i.e., 3.5 cm diameter Petri dishes) and MTT assay for small scale set-up (i.e., 96 well plates). Since MTT assay cannot be strictly considered a test to evaluate cell replication, an initial set of experiments were performed in Petri dishes and cells were counted in a Burker chamber, evaluating both cell number and cell viability by Trypan blue staining. Culture of ADMSCs in the 3D environment resulted in an increased cell number by direct count (see results reported below) and increased MTT signal, thus validating the use of this assay as a test to analyze the growth of ADMSCs in different conditions.

### 3.2. ADMSCs Growth inside 3D Fibrin-Derived Matrix Versus 2D Plastic Surface: Direct Cell Count

**Figure 1:**
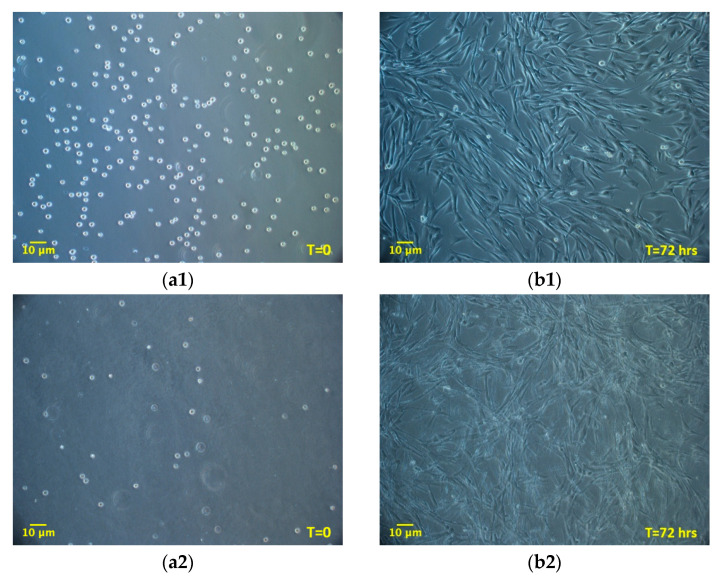
Morphology of adipose tissue derived mesenchymal stem cells (ADMSCs) cultured on plastic surface (**a1**, **b1**; 100×) or embedded in the 3D fibrin-based matrix (**a2**, **b2**; 100×). When seeded on plastic surface ADMSCs take on the classic fibroblast-like shape (**b1**, 72 h). When maintained inside the 3D matrix, cells are dispersed homogeneously and exhibit a thinner spindle-like morphology (**b2**, 72 h).

**Figure 2:**
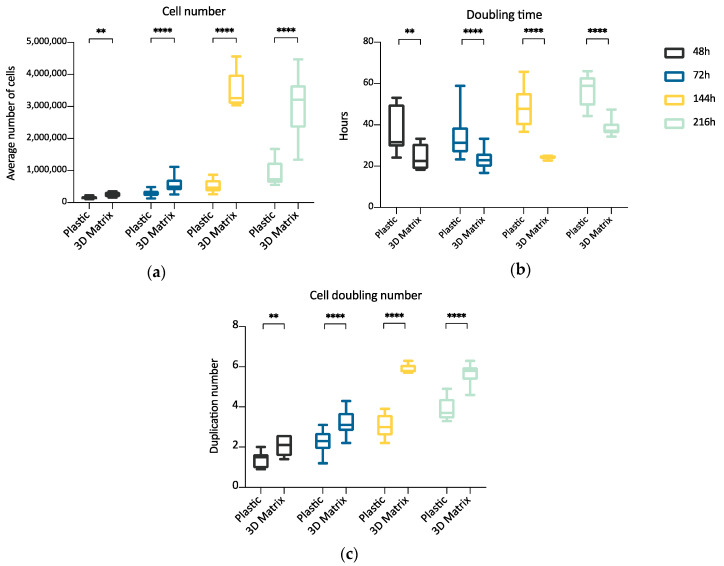
Box plot illustrating differences in average cell number (**a**), cell doubling time (**b**), and cell doubling number (**c**) obtained from cells maintained on plastic surface or inside a 3D matrix prepared from PPP, at different time points (48, 72, 144, 216 h) (n = 5). Statistically significant differences are indicated as follows: ** *p* ≤ 0.01, **** *p* ≤ 0.0001.

The experiment compared ADMSCs growth, by direct cell count, on a canonical 2D plastic surface and inside a 3D matrix obtained by jellying PPP. The aim was to assess if a 3D fibrin-based matrix is suitable to expand ADMSCs and can improve cell’s growth rate. ADMSCs (P3-P4) showed a roundish shape once seeded in both supports (Figure 1a1,a2). On plastic, the cells adhered in few hours, resuming their typical fibroblast-like shape (Figure 1b1), while inside the 3D matrix the cells were dispersed in different layers, taking a thinner fibroblast-like appearance (Figure 1b2). The jellification time of the matrix (within a mean of 5–7 min) always allowed a homogeneous distribution of cells. After a mean of 72 h from seeding, ADMSCs reached a confluence of about 80–90% on plastic, maintaining their characteristic fibroblast-like morphology. Cell growth was also evident inside the 3D fibrin-based matrix, although the cells’ confluence was not determinable in this environment (Supplementary Material, Video S1). Cell count was performed at 48, 72, 144, and 216 h. Although 144 and 216 h are extended time points not suitable for conventional cell growth on plastic surface, they were chosen to evaluate cell growth inside the 3D environment, since the larger available space and the different environment could maintain cell replication for an extended time. Starting from 48 h, there was a significant difference (*p* < 0.01) between the average cell number in the two environments (Table 1, Figure 2a), with a higher number of cells obtained in the 3D environment. Average DT was significantly lower (*p* < 0.01) for cells grown inside the matrix (24.4 ± 6.2 versus 37.7 ± 11.1 for cells maintained on plastic surface). Correspondingly, average CDn was higher for 3D grown cultures (2.1 ± 0.5 versus 1.4 ± 0.4). Also, at 72 h after seeding, the average cell number was higher (*p* < 0.0001) inside the 3D matrix in comparison to the 2D culture environment: the number of ADMSCs was more than twice when cultured in the 3D environment (Figure 2a; Table 1). At the same time point, DT was 23.6 ± 4.9, corresponding to a CDn of 3.2 ± 0.7 for cells grown in 3D, while it was 34.3 ± 9.8 with a CDn of 2.2 ± 0.6 for the same cells grown on plastic surface (*p* < 0.0001 for both values) (Figure 2b,c; Table 1). Furthermore, after 144 h, in the 3D matrix, the cell population was about seven times higher than ADMSCs grown on plastic (*p* < 0.0001), (Figure 2a; Table 1). With regard to the DT, this parameter increased for the cells on the plastic surface, indicating a slow proliferation rate (48.5 ± 9.2), but decreased for those included in the 3D matrix (24.3 ± 0.9), indicating a higher replicative capacity of cells embedded in this environment (Figure 2b, *p* < 0.0001). Box plot graphs were used to evaluate the distribution of experimental data at different time points. Box plots showed that, after 144 h, doubling time and cell-doubling number for the 3D matrix had a low dispersion within the different experiments. Moreover, the cell doubling number in the 3D environment was about two times higher than that of cells grown on plastic (5.9 ± 0.2 vs. 3.1 ± 0.6; *p* < 0.0001) (Figure 2c; Table 1). Relative to the last experimental point (216 h, 9 days of culture), the number of cells grown on plastic increased to a limited extent (CDn 0.7 ± 0.6) compared to that at 144 h of culture (Figure 2a). On the other hand, the number of cells grown inside the matrix was lower with respect to the 144 h-time point, indicating that the cells did not replicate further. Nevertheless, there was a statistically significant difference with regard to the average cell number (*p* < 0.0001), DT (*p* < 0.0001), and CDn (*p* < 0.0001) between the two culture environments.

### 3.3. ADMSCs Growth in PRP-Derived 3D Matrix Versus PPP-Derived 3D Matrix: Direct Cell Count

**Figure 3:**
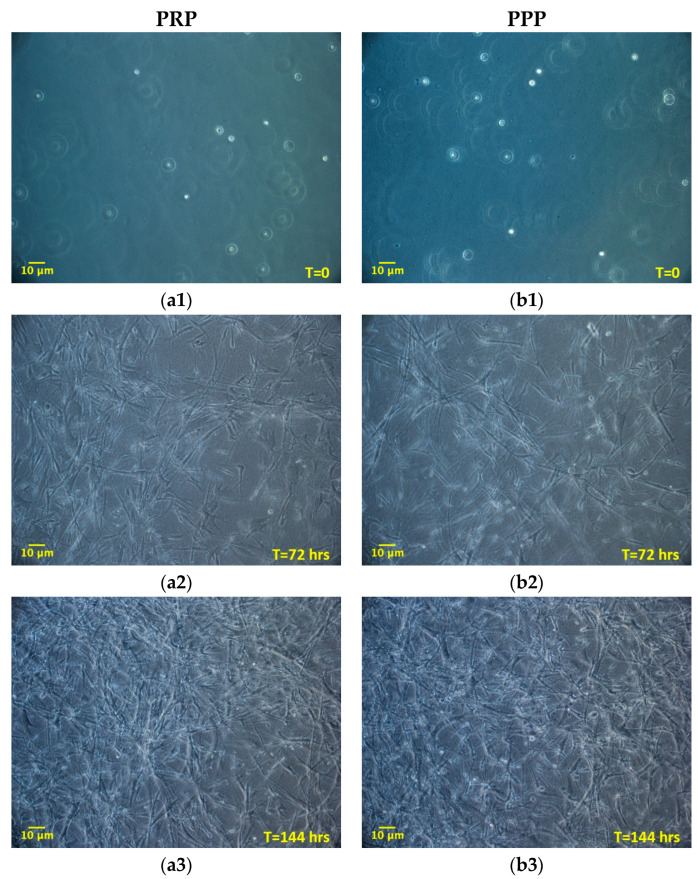
Morphology of ADMSCs grown inside a 3D matrix prepared from platelet-rich plasma (PRP) (**a1**, **a2**, **a3**; 100×) or PPP (**b1**, **b2**, **b3**; 100×), at different time points (0, 72, 144 h). The initial round-shaped morphology (a1, b1; time = 0 h), is changed in a spindle, fibroblast-like morphology at 72 and 144 h (**a2**, **b2**, **a3**, and **b3**). Cells gradually populate the whole 3D fibrin-derived scaffold, spreading over the entire thickness. No morphological differences are observed between cells maintained in PRP or platelet-poor plasma (PPP) derived matrix.

**Figure 4:**
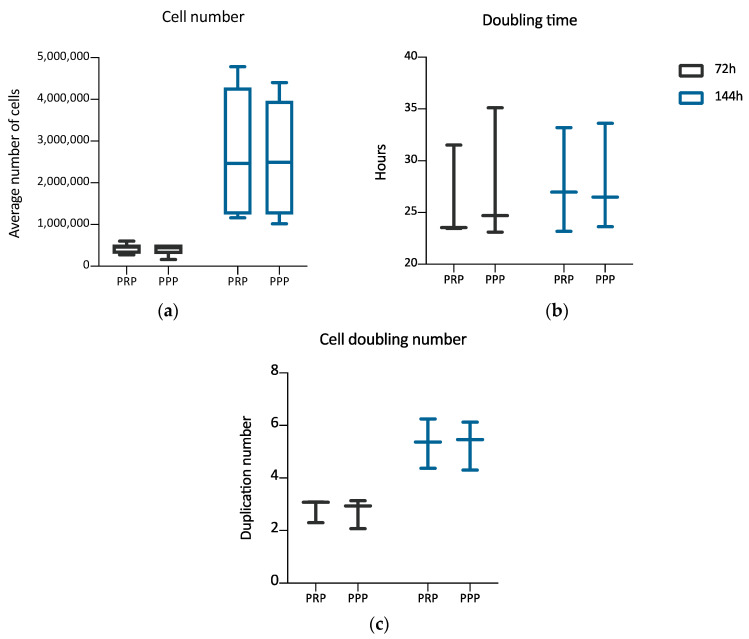
Box plot illustrating differences in average cell number (**a**), cell doubling time (**b**), and cell doubling number (**c**) of cells maintained inside a 3D matrix prepared from PRP or PPP, at different time points (72, 144 h) (n = 3). No statistically significant differences are observed between the two culture conditions.

Once established the positive effect of the 3D environment prepared using PPP on the ADMSCs growth, a further set of experiments, aimed at verifying a possible different behavior of cells grown inside 3D environments derived from PPP or PRP, was performed. The aim was to evaluate if the greater amounts of growth factors contributed by PRP in comparison to PPP could modify the cell growth inside the 3D environment. Two different time points were considered: 72 and 144 h. Figure 3 shows the cultures’ appearance at these time points. Both at 72 and 144 h, the average number of cells was similar between the two types of 3D environment, without a statistically significant difference (Table 2). The DT and the CDn were also not significantly different, both at 72 and 144 h. The box plot analysis, at each experimental point, highlights the uniformity of behavior between ADMSCs populations in the two different culture conditions. Variability was observed considering the number of cells obtained in matrices in the three different experiments. However, no significant differences could be found between PRP and PPP (Table 2; Figure 4a–c).

### 3.4. SVF Cells Growth in 3D Fibrin-Based Matrix Versus 2D Plastic Surface: Direct Cell Count

**Figure 5:**
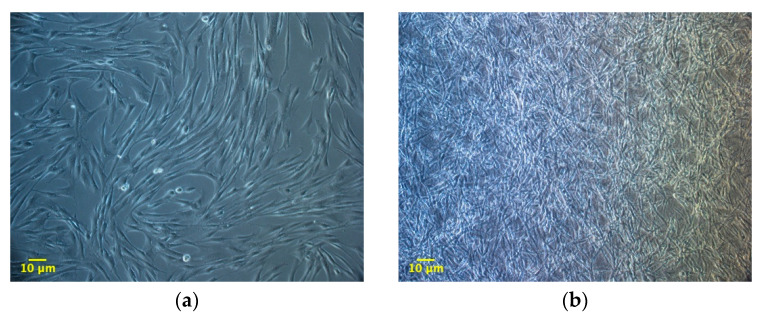
Morphology of adipose tissue derived stromal vascular fraction (SVF) cells (100×), expanded on the plastic surface (**a**) or within a 3D matrix prepared from PPP. (**b**) The cells were expanded until they reached about 90% of confluence on plastic (average 192 h). The morphology resembles what observed for ADMSCs cultures in the two different environments.

**Figure 6:**
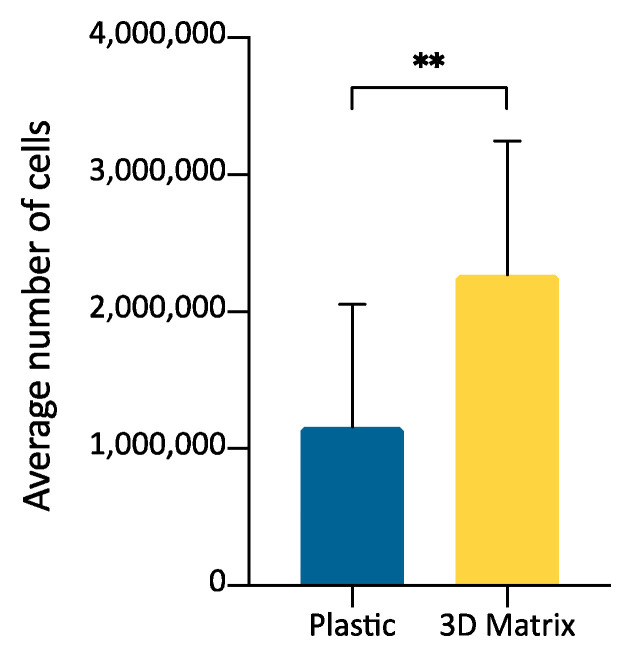
Graph bars representing the average number of cells obtained from adipose tissue derived SVF cells expanded on plastic surface or inside a 3D matrix prepared from PPP (*n* = 3). Statistically significant differences are indicated as follows: ** *p* ≤ 0.01.

The growth of SVF cells obtained by collagenase digestion of adipose tissue was compared by culturing cells in a 3D matrix obtained from PPP jellification or on plastic surface. The purpose was to evaluate if the SVF could be expanded after seeding inside the fibrin-matrix and to evaluate ADMSCs replication in the two environments. Cell count was performed when cells on plastic surface reached about 90% confluence (average, 192 h) (Figure 5). 3D fibrin-based matrix showed a mean increase in the number of cells of 2.49 times compared to the plastic surface (Table 3). The difference in the number of cells obtained within the 3D matrix compared to the 2D plastic surface was statistically significant (Figure 6, *p* < 0.01).

### 3.5. ADMSCs Growth in 3D Fibrin-Based Matrix Versus Plastic Surface: MTT Assay

**Figure 7:**
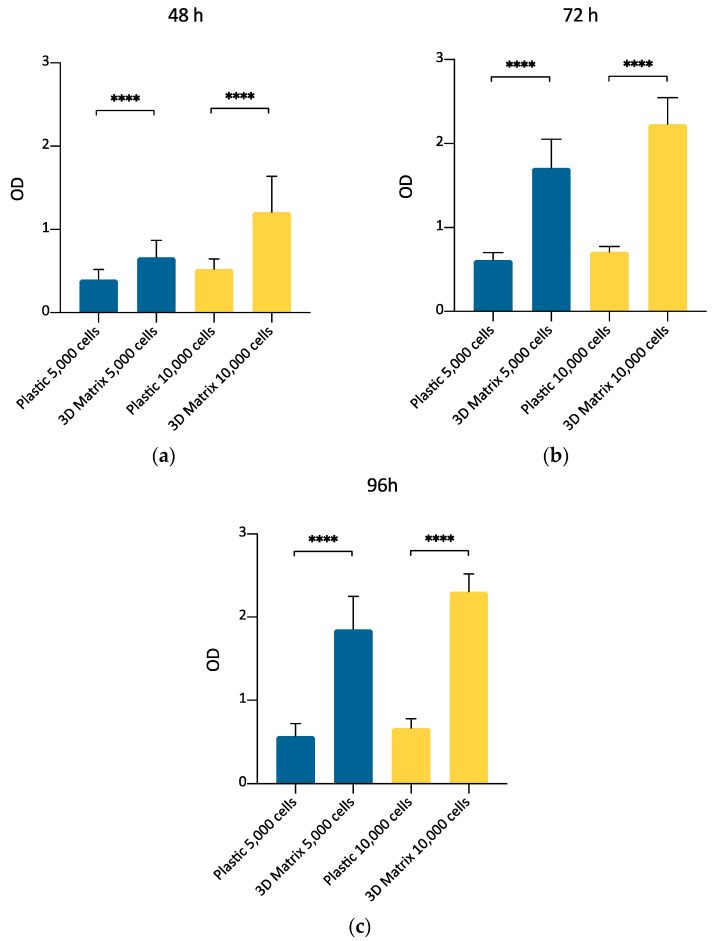
Evaluation of ADMSCs growth on plastic surface or inside the 3D fibrin-based matrix prepared from PPP, by MTT assay at 48 (**a**), 72 (**b**), and 96 (**c**) hours. The number of cells seeded was 5000/well (blue bars) or 10,000/well (yellow bars). The metabolic MTT assay confirmed the results observed by direct cell count. 3D fibrin-based matrix is a suitable environment to stimulate ADMSCs’ growth. Statistically significant differences are indicated as follows: **** *p* ≤ 0.0001.

This experimental set aimed to compare the growth of canine ADMSCs cultured on a canonical 2D plastic surface with that of the same cells cultured inside a 3D matrix obtained by gelling PPP in 96-well plates. The purpose was to confirm, by MTT assay, that 3D matrix is a suitable environment to support ADMSCs replication, thus making plausible exploring further culture conditions to prepare cells suitable for clinical applications. A comparison between MTT assay performed at three different time points (48, 72, and 96 h) indicated (Figure 4a) that ADMSCs actively proliferated inside a 3D matrix prepared with PPP, as already found by direct cell count (Section 3.2). Their replication rate was higher compared to the cells cultured on plastic, both after 48, 72, and 96 h of culture (Figure 7; *p* < 0.0001). MTT assay results were confirmed for both densities of 5,000 and 10,000 cells plated per well. This experimental set also confirmed that MTT assay can be considered a valuable method to determine ADMSCs growth inside the 3D matrix.

### 3.6. ADMSCs Growth on Plastic Surface and within a 3D Fibrin-Based Matrix: Comparison between FBS and Allogeneic Canine Serum by MTT Assay

**Figure 8:**
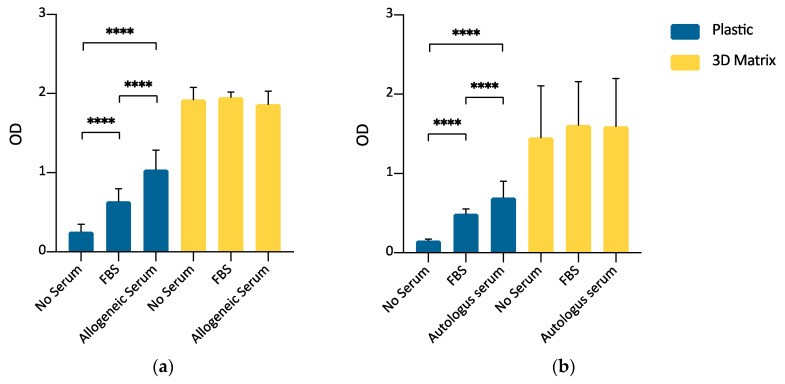
(**a**) Comparison between FBS and allogeneic canine serum as a medium supplement for ADMSCs culture, by MTT assay (5000 cells/well; 72 h). When seeded on plastic (blue bars), supplementation of medium with 10% allogeneic serum induced a statistically significant stimulus to cell growth with respect to FBS. When cells were cultured inside a 3D fibrin-based matrix prepared from PPP (yellow bars), allogeneic serum, and FBS supplementation, or no supplementation in the culture medium, did not induce different cell growth. Statistically significant differences are indicated as follows: **** *p* ≤ 0.0001 (n = 3). (**b**) Autologous serum as a substitute for FBS in medium supplementation for ADMSCs growth (5000 cells/well; 72 h). When cells were cultivated on plastic surface (blue bars), 10% autologous serum medium supplementation was an effective substitute for 10% FBS supplementation. When cells were seeded inside a 3D fibrin-based matrix (yellow bars), no serum supplementation, FBS or autologous serum supplementation induced comparable results. Statistically significant differences are indicated as follows: **** *p* ≤ 0.0001 (*n* = 3).

Once verified the ability of the 3D fibrin-based matrix to support the ADMSCs growth by direct cell count and MTT assay, a further experimental set was carried out to evaluate the possibility of expanding canine ADMSCs avoiding the supplementation of culture medium with FBS, thus reducing the risks of adverse immunological reactions when ADMSCs are used in therapeutic applications. As reported in the material and methods section and shown by Figure A1, FBS supplementation was not used for the preparation of the 3D environment, but only as a supplement in the formulation of the liquid medium layered above the gel. This experimental set-up compared the cells’ growth in serum-free medium with growth in the presence of 10% FBS and 10% allogeneic serum derived from PPP in the liquid culture medium, both on the plastic surface and inside the 3D matrix. For 3D cultures, supplementation was limited to the overlying medium only. ADMSCs seeded on plastic (2D culture) demonstrated a higher growth rate when cultured in the presence of either 10% FBS or allogeneic canine serum with respect to serum-free medium (*p* < 0.0001). Furthermore, the supplementation of growth medium with 10% of allogeneic canine serum demonstrated a statistically significant enhancement of cell replication with respect to 10% FBS (*p* < 0.0001). When the cells were seeded inside the 3D fibrin-based matrix prepared with canine PPP, there were no statistically significant differences in cell growth between the serum-free medium and media supplemented with 10% FBS or 10% allogeneic canine serum. (Figure 8a).

### 3.7. ADMSCs Growth on Plastic Surface and inside a 3D Fibrin-Based Matrix: Comparison between FBS and Autologous Serum by MTT Assay

The experiment aimed to assess if autologous serum can be used as FBS substitute in the expansion of ADMSCs. The use of autologous blood-derived products avoids the risks associated with a xenobiotic supplement (FBS) and increases the safety of the cell preparation, avoiding the risk of the transmission of infectious diseases. Canine ADMSCs were expanded in medium supplemented with 10% autologous serum or FBS. Autologous serum could replace FBS, both for 2D and 3D cultures. A statistically significant increase of the signal was observed in this set of experiments for the cells cultured in autologous serum (Figure 8b, *p* < 0.0001) but only on the plastic surface.

### 3.8. Effect of Allogeneic PL Versus FBS on ADMSCs Growth on Plastic Surface, MTT Assay

**Figure 9:**
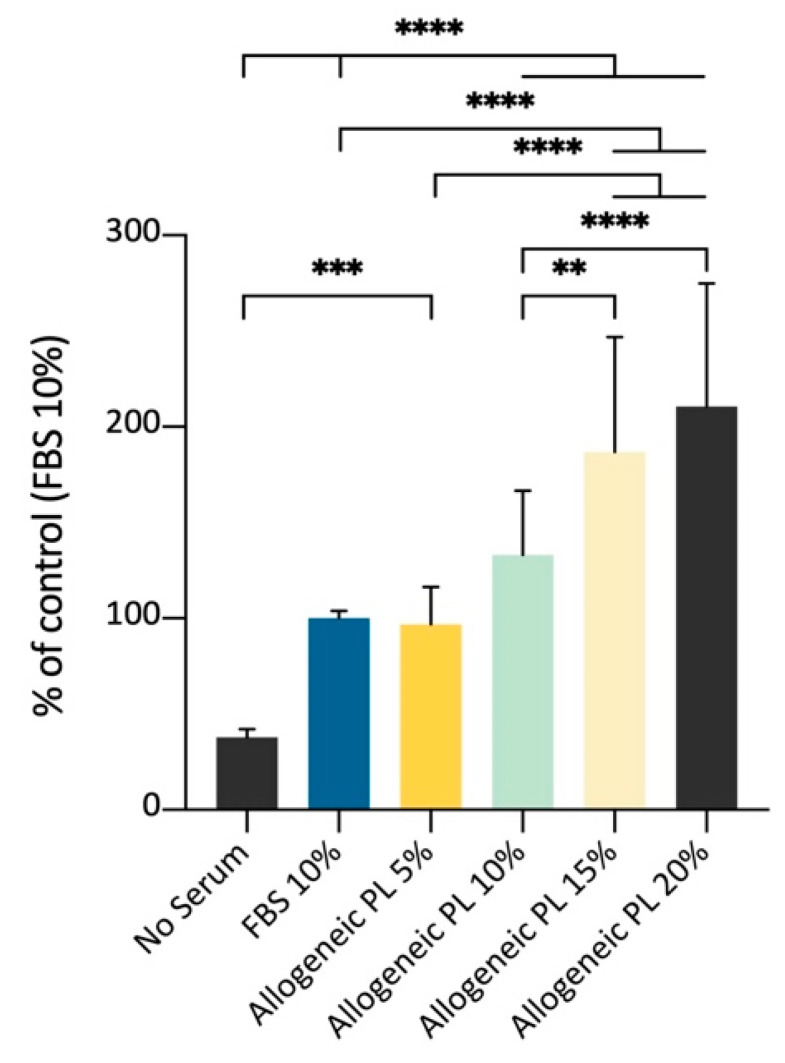
Effects of different percentages of allogeneic PL medium supplementation for the culture of ADMSCs on plastic surface, MTT assay (5000 cells/well; 72 h). Allogeneic PL represents an effective stimulus for cell growth, substituting 10% FBS medium supplementation. Statistically significant differences are indicated as follows: ** *p* ≤ 0.01, *** *p* ≤ 0.001, **** *p* ≤ 0.0001 (*n* = 3).

Following the abovementioned results, the ADMSCs’ growth on plastic was evaluated using as medium supplement different amounts of PL prepared from allogeneic blood. The aim was to confirm the possibility to support cell growth with a culture medium supplemented with different percentages of an allogeneic but species-specific preparation enriched with growth factors released by platelets, in substitution of the FBS, thus improving cells’ safety. The spectrophotometric MTT assay showed an increased signal as a function of growing PL volume percentages, until the maximum value tested (20%). The results of this experimental set demonstrated that PL can be used to amplify canine ADMSCs in vitro, with a growth stimulus comparable to the supplementation with 10% FBS (Figure 9).

### 3.9. ADMSCs Growth on Plastic Surface: Comparison between Allogeneic Serum, Autologous Serum, and FBS by MTT Assay

**Figure 10:**
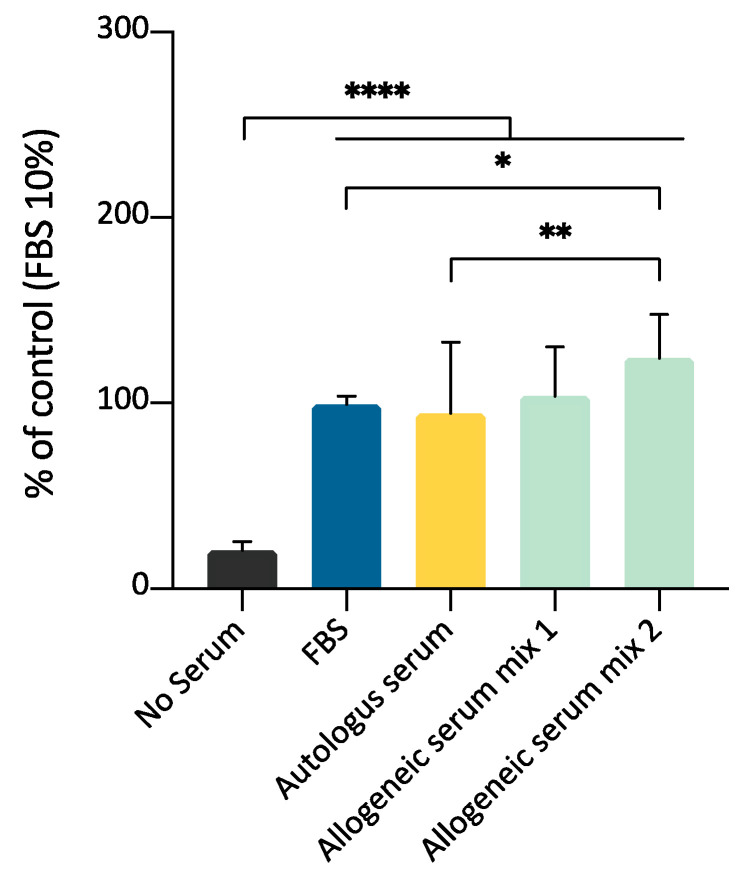
Comparison between allogeneic serum, autologous serum, and FBS as medium supplements for ADMSCs growth, by MTT assay (5000 cells/well; 10% supplementation; 72 h). Both autologous serum and allogeneic serum mix supplementation can substitute FBS for ADMSCs culture. Statistically significant differences are indicated as follows: * *p* ≤ 0.05, ** *p* ≤ 0.01, **** *p* ≤ 0.0001.

In this experimental set, the purpose was to compare the effect of allogeneic and autologous serum on the expansion of ADMSCs; the effectiveness of both species-specific blood-derived products were compared to FBS supplementation. The effects of 10% supplementation of culture medium with allogeneic serum, autologous serum, or FBS were evaluated on three different ADMSCs preparations. Two different allogeneic serum mix were prepared using serum from three different animals for each mix, to evaluate a possible batch variability. MTT assay demonstrated that both allogeneic and autologous serum could stimulate cell growth. A significant statistical difference was observed between autologous serum and mix 2, but not mix 1 (Figure 10). This experimental set confirms that canine serum, either autologous or heterologous, is a suitable substitute for FBS for canine ADMSCs growth.

### 3.10. Phenotypic Characterisation of Cell Cultures

**Figure 11:**
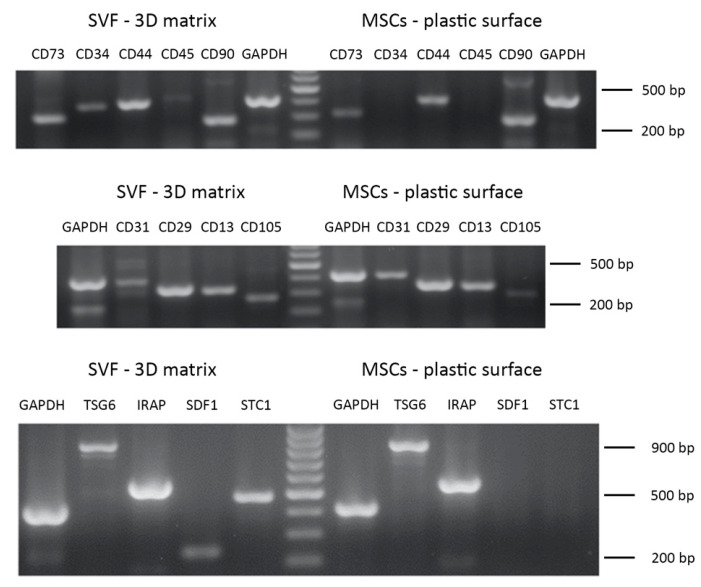
Phenotypic characterization of ADMSCs and adipose tissue-derived SVF by RT-PCR. Upper and middle panel: gene expression analysis for MSCs markers. Lower panel: expression analysis of genes involved in anti-inflammatory and immunomodulatory features of MSCs.

The phenotypic characterization of ADMSCs (P3) expanded on the plastic surface performed by RT-PCR showed cell positivity to the following markers, typical of MSCs: CD13, CD29, CD44, CD73, CD90, CD105 (Figure 11, Table 4) [16,32]. On the other hand, these cells were negative, as expected, to the expression of the CD45 hematopoietic marker and CD34. The cells showed a faint positivity to the endothelial marker CD31 (Table 4). When ADMSCs were maintained inside the 3D fibrin-based matrix, they also expressed CD34. The phenotypic characterization was also extended to a series of markers involved in the modulatory effects on inflammation exerted by MSCs: TNF-stimulated gene 6 protein (TSG-6), IL-1 receptor antagonist (IRAP), stromal cell-derived factor 1 (SDF-1), stanniocalcin-1 (STC-1) (Table 4, inflammation modulators). ADMSCs expressed TSG6 and IRAP when cultured on plastic surface, while they also expressed SDF-1 and STC-1 when grown inside the 3D scaffold. An analysis of gene expression was also performed on SVF cells cultured inside the 3D fibrin matrix. These cells demonstrated a pattern of expression of marker genes similar to that described for ADMSCs, except for CD45 and CD34, which were expressed by SFV cells grown inside the 3D matrix. Regarding TSG-6, IRAP, SDF-1, STC-1, all genes were expressed (Table 4, inflammation modulators) by SVF cells.

### 3.11. ADMSCs Differentiation

**Figure 12:**
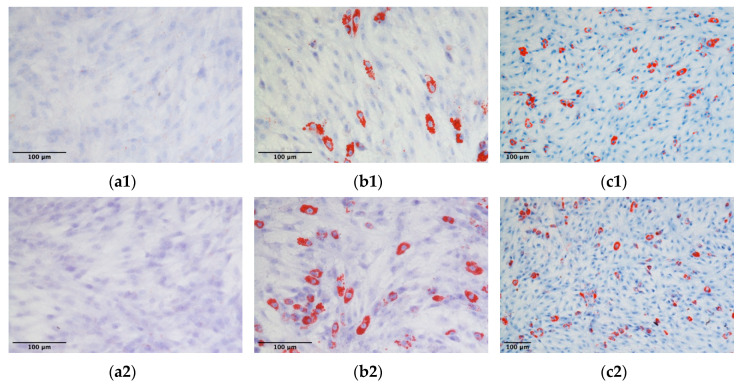
Adipogenic differentiation of ADMSCs: ADMSCs expanded on plastic (**b1**,**c1**) or inside the 3D fibrin-based matrix (**b2**,**c2**) were positive to Red Oil O staining after stimulation with adipogenic differentiation medium. Control unstimulated cells (**a1**,**a2**) did not accumulate lipid droplets.

The capacity of ADMSCs to undergo differentiation towards the adipocyte and osteocyte lineages was confirmed for three different ADMSC populations expanded on a 2D plastic surface or inside the 3D fibrin-based matrix. No lipid droplets accumulation was observed in unstimulated control cultures (Figure 12a1,a2). On the contrary, cell cultures derived from 2D plastic or 3D fibrin-based environment, stimulated with adipogenic differentiation media demonstrated the presence of Red oil O stained cells (Figure 12b1,c1,b2,c2). The count of differentiated cells (i.e., cells positive to Red Oil O staining) demonstrated that the mean percentage of differentiated cells was 17.7 ± 4.8% for cells expanded on the plastic surface, and 17.2 ± 3.6% for ADMSCs expanded in the 3D environment. No significant differences were observed between the adipogenic potential of the two cell populations.

**Figure 13:**
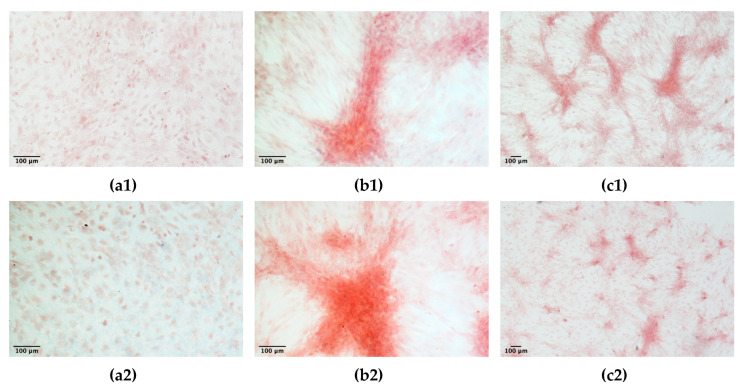
Osteogenic differentiation of ADMSCs: ADMSCs expanded on plastic (**b1**,**c1**) or inside the 3D fibrin-based matrix (**b2**,**c2**) were positive to Alizarin Red staining after stimulation with osteogenic differentiation medium. Control, unstimulated cells (**a1**,**a2**) were negative to staining.

Osteogenic differentiation was demonstrated by a positive Alizarin Red staining in cultures treated with osteogenic medium. Both cells grown on plastic surface (Figure 13b1,c1) and grown inside the 3D matrix (Figure 13b2,c2) were positive; on the contrary, control unstimulated cultures were negative to Alizarin Red staining (Figure 13a1,a2).

## 4. Discussion

The application of MSCs in veterinary regenerative medicine has attracted widespread interest in the last years. Although a substantial consensus exists on their clinical potential, much remains unclear about their real therapeutic properties and effectiveness [12,33,34]. As a matter of fact, despite the knowledge about biological and therapeutic features of these cells has strongly improved in the last decade, for most diseases the proper cell preparation procedures, as well as the best treatment strategies, have not been determined yet. From this point of view, the key issues to be addressed concern, for example, the choice of MSCs tissue source, the in vitro procedures applied to obtain their efficient and safe amplification, the therapeutic protocols used for their clinical application (i.e., which route of administration, which number of cells, how many administrations, etc.) [34]. A review of the literature highlights that different cell sources are used, cells are expanded in vitro following different protocols, and different dosages and timetables are reported for the treatment [34,35].

The in-vitro expansion of MSCs is a relatively simple task for the animal species of interest in veterinary medicine. Nevertheless, although MSCs easily adhere to plastic showing active replicative activity, their culture usually requires the use of FBS as a growth-supporting supplement. Furthermore, their therapeutic application in diseases that need a systemic route for the delivery to the target tissue requires the availability of a large number of cells and, consequently, an extended period of in vitro expansion for their preparation [10,11,21,36,37]. As a consequence, the use of expanded MSCs in veterinary regenerative medicine requires the availability of safe cell preparations and, possibly, an optimization of the timing of their application. Both points are crucial for a safe and effective therapy.

The present work aimed to investigate the in vitro expansion of canine ADMSCs by evaluating how different culture environments can influence cell replication, and hence the time interval between tissue sample collection and ADMSCs delivery to the patient. In particular, it was assessed whether FBS could be substituted by canine blood-derived supplements, thus improving cell safety by eliminating xenobiotic components from the culture medium. Furthermore, it was evaluated whether cell culture in a three-dimensional (3D) environment based on a fibrin network can increase the replicative efficiency of both ADMSCs and adipose tissue SVF derived cells. SVF prepared by digestion of adipose tissue has been suggested as an alternative to ADMSCs in regenerative medicine, since it is composed of a heterogeneous cell population featuring pro-regenerative properties, is more easily prepared and does not need extensive in vitro expansion: SVF can be used in-vivo, as a point-of care approach, with a minimal manipulation to promote tissue healing [15].

For this purpose, various substitutes for FBS were explored, and in particular: canine homospecific serum (prepared from PPP), PRP, and PL. Moreover, homospecific plasma obtained by mixing samples prepared from different animals and autologous plasma prepared from the same donor of the cells were compared to evaluate their growth-promoting activity. The growth environment for ADMSCs expansion was the plastic surface used for traditional two-dimension (2D) cell cultures or, alternatively, a 3D environment obtained from the polymerization of the fibrinogen contained in the platelet-poor plasma and the different platelet concentrates analyzed.

The stimulatory effects of the 3D fibrin-based matrix observed on the in vitro expansion of ADMSCs (Table 1, Figure 2) support the use of this 3D environment to expand high numbers of canine ADMSCs in a short time. In 48 and 72 h cultures, the 3D fibrin matrix allows for an efficient replication of ADMSCs, ensuring the production of a higher cell number in comparison to plastic surface environment. DT for cells grown inside the matrix is lower than the one observed on plastic, thus reaching a higher CDn at both time points. It is noteworthy that the DT remains constant for both environments, demonstrating the active replication of ADMSCs. Furthermore, in the 3D environment the DT remains constant until 144 h, thus suggesting that this culture set-up is suitable to prepare of a large number of actively proliferating ADMSCs.

Cell growth assessment by direct cell count in 3D fibrin-based matrix prepared with PPP or PRP demonstrated that ADMSCs’ growth was not affected by the nature of the 3D fibrin matrix. Indeed, PPP and PRP derived matrix demonstrated a similar capacity to stimulate cell growth (Table 2, Figure 4). These results indicate that, at least in our experimental set-up, a fundamental support for cell growth is probably provided by the 3D fibrin matrix itself, rather than by the platelet-derived trophic factors. Alternatively, the bioactive molecules supporting cell expansion, which are already present in sufficient amount in the environment provided by PPP and trophic factors derived by platelets, do not apport further growth stimuli.

These results could have interesting implications both for the laboratory preparation of cells and the clinical practice. The first observation is that the fibrin-based 3D environment could provide a significant contribution to the preparation of large numbers of cells needed in some clinical set-ups, requiring, for example, systemic administrations. Although a clear consensus does not exist, a mean of 0.5–2.0 ×10^6^ cells/kg have been used in the treatment of systemic or organ diseases in the dog and cat [21,22]. The expansion of several millions of cells could be time-demanding, thus precluding the adequate timing of the therapy; therefore, the high cell number produced in a relatively short time by using a 3D fibrin environment could aid to overcome the critical point of quick availability of an adequate amount of ADMSCs to be employed in the clinical practice. Furthermore, the availability of a 3D matrix able to maintain cells viability and growth could be of interest in clinical application, both in soft and hard tissue lesions, where the 3D matrix seeded with ADMSCs could be applied to improve healing, providing an expanding population of active cells in-vivo.

A further comparison of growth supplements and environments suitable to expand ADMSCs was performed. These experiments confirmed that the 3D matrix is a potent stimulus to cell replication. Canine serum, used to prepare the fibrin-based matrix, strongly induced cell proliferation (Figure 8a), suggesting that canine ADMSCs can be grown in 3D fibrin-based matrices without the need for xenogeneic components and that the use of PRP, PL, or PPP-derived serum is equivalent.

Interestingly, the substitution of xenogeneic supplements (FBS) by dog plasma derivatives (i.e., allogeneic and autologous serum) for the expansion of ADMSCs on the traditional plastic surface provided an effective stimulus for cell growth (Figure 7 and Figure 8). Taken together, these results suggest that plasma-derived supplements can be a useful tool to improve the efficacy and safety of the preparation of ADMSCs, especially when a large number of cells is required for therapeutic application.

The in vitro preparation of MSCs from adipose tissue samples routinely requires collagenase digestion as an initial step to obtain the so-called SVF, a heterogeneous cellular fraction from which, by sequential passages in culture, the ADMSCs population is selected [15]. Although the latter is the most frequently used cell population for therapeutic applications, SVF itself has an acknowledged therapeutic significance and is often used, without further amplification and in vitro passages, both in humans and domestic animals [38,39]. Our results suggest that seeding the SVF inside the 3D matrix could be effective for faster cell expansion (Table 3, Figure 6) and point of care, acute clinical application.

The FBS is by far the most widely used supplement for the growth of primary cells in vitro. It provides a complex mix of biological factors that contribute to maintaining vitality, biological functions, and that promote the replication capacity in cells isolated from living tissues seeded in an artificial environment, characterized by unusual molecular signals and adhesion surfaces. Although essential for most primary cultures, FBS represents a potential risk: it can be a vehicle of pathogens and could cause immunological responses, ranging from insignificant to highly significant from a clinical point of view, in patients treated with cell therapy [40]. Furthermore, it is not clear if a xenogeneic serum might have effects on the biological activity of in vitro cultured cells. This is a key point dealing with the in vitro culture of cells to be applied in cell therapy. In principle, the use of homospecific cell-depleted supplements should reduce the risk of immune responses in transplanted patients. According to this, in the last decade, in human medicine the expansion of MSCs in serum-free media, with strictly controlled chemical composition, or, alternatively, of media supplemented with platelet concentrates of human origin, has almost become a standard [19,23]. This same subject represents a more complex issue in veterinary medicine, where the availability of enhanced media or chemically defined supplements dedicated to individual animal species, including dogs and horses, is extremely limited. The use of FBS for MSCs expansion is therefore still the routine in veterinary medicine for dog cell cultures. The data presented in this work allow hypothesizing an effective use of homospecific, allogeneic, or autologous serum and platelet concentrates as substitutes for FBS for the supplementation of culture media to be applied in regenerative medicine protocols based on the use of canine ADMSCs. The preparation of serum, PRP, or PL can be obtained from whole venous blood taken from the patient at the same time as the sample of the adipose tissue collected for the isolation of ADMSCs. In fact, the stimulus to cell replication provided by these supplements is comparable to that of the FBS, but this approach strongly reduces the risk of immunological reaction in the patient after cell administration.

Moreover, our results point out that homospecific plasma derivatives can also be used for the expansion of ADMSCs in 3D environment obtained from the polymerization of fibrinogen. Matrices prepared from plasmatic fibrinogen represent a scaffold for tissue regeneration that is receiving strong interest in regenerative medicine [27,28]. The 3D network of fibrin nanotubes that is produced following blood coagulation represents a physiological support for tissue repair and plays a primary role in coordinating cellular activity in the healing of damaged tissue. It organizes a reservoir of signal molecules released by the cells involved in the inflammatory process at first, and in the regenerative phase of tissue healing later. In addition, it provides physical support to the migration of the reparative cells themselves [41,42]. In this work, we evaluated whether the 3D matrix obtained by in vitro polymerization of PPP, PRP, and PL is a valid support for the in vitro expansion of ADMSCs. Both canine PPP, PRP, and PL-derived fibrin matrix can support the expansion of ADMSCs and SVF cells in substitution of xenobiotic FBS. The availability of a semi-solid support for the growth of ADMSCs that can be employed for therapeutic purposes has a double advantage. First, the rate of replication of ADMSCs sharply increases compared to the regular plastic surface used for traditional 2D cultures, thus allowing to obtain higher numbers of cells suitable for the therapeutic application, in the absence of xenobiotic supplements. This is a significant advantage for systemic application where a large number of cells is required. The second advantage of 3D cultures is that the fibrin scaffold and the population of cells grown therein can be easily manipulated by the clinician and directly applied in point-of-care therapeutic applications such as the treatment of skin wounds, or in the case of soft-tissue lesion accompanied by losses of tissue. Interestingly, both PRP and SVF application have been proposed in recent years to improve wound healing [43,44,45]. Yin et al. proposed that the synergistic effects of PRP and SVF promote a safe and effective wound healing in chronic ulcers in humans [46]. Our data suggest that the combination of the two regenerative approaches can be accomplished in the dog. Both SVF and platelet concentrates-based therapeutic could be prepared just after tissue and blood sample collection; furthermore, SVF cells could be maintained and expanded in vitro in a 3D fibrin-based matrix for subsequent applications.

To compare the phenotype of ADMSCs grown on a plastic surface and inside the 3D fibrin scaffold, and to characterize SVF cells grown inside the 3D scaffold, a panel of markers has been analyzed by semi-quantitative RT-PCR. Both ADMSCs populations expressed CD29, CD90, CD73, CD44, CD13, CD31, and CD105. The cells were negative for CD45. CD34 was expressed only by ADMSCs grown in the 3D environment. A similar pattern was observed for SVF cells, although they showed a faint expression of CD45, as already reported for this heterogeneous cell population that contains, for example, CD45+ macrophages [15]. SVF cells also expressed CD34. CD34 expression in ADMSCs is a matter of investigation and discussion by the scientific literature. Although CD34 gene is expressed by hematopoietic progenitors and endothelial cells, and is not usually expressed in MSCs, different authors report its expression in ADMSCs in several animal species [32,43]. Dykstra et al. suggest that its expression in MSCs can be lost as a consequence of in vitro culture [43]. As a matter of fact, in our cultures, CD34 was expressed only in cells grown within the 3D fibrin scaffold, and its expression could reflect a cell response to different environmental cues. Moreover, CD34 expression in SVF cells has already been reported, because of the presence of macrophages, endothelial precursor cells, and pre-adipocytes [15]. Furthermore, we analyzed the expression of markers that, although not essential to determine the phenotype of MSCs, are related to their biological activity [47]. Interestingly, ADMSCs expressed in both environmental conditions TSG-6 and IRAP. Both TSG-6 and IRAP although using different pathways, contribute to the anti-inflammatory and immunomodulating activity of [48,49]. Furthermore, SVF expressed SDF-1 and STC-1 when maintained in 3D environment. SDF-1 is a key player in the recruitment of MSCs and other progenitor cells in damaged tissues [50]. STC-1 has a protective role against reactive oxygen species (ROS) and a potential anti-inflammatory action [51]. Altogether, these data support the use of the 3D environment to maintain key biological features of canine ADMSCs as suggested by others for human MSCs [48].

Finally, both ADMSCs grown on plastic and inside the 3D matrix demonstrated the ability to differentiate towards the osteogenic and adipogenic lineages, providing a further support to the feasibility to expand ADMSCs inside a fibrin-based 3D matrix.

The work has some weak points that need to be addressed in the future. We are aware that to demonstrate that the use of a 3D fibrin-based matrix to expand ADMSCs for their clinical application is safe and feasible an extensive cell characterization is required, that our work only partially provides. To this aim, a cytofluorimetric analysis of cells grown inside the 3D matrix and their comparison to cells grown on the traditional plastic dishes should be performed. Phenotypic characterization by cytofluorimetry of MSCs should be indeed reinforced by investigating other cellular parameters affecting their therapeutic function; these might include cell senescence and production and content of microvesicles. Indeed, several studies performed using platelet concentrates to expand in vitro human MSCs, have demonstrated that these blood-derived products do not alter cell phenotype, thus supporting their use for expansion of clinical-grade cells [24,52]. On the contrary, data related to canine MSCs are quite limited [25] and, in particular, to our knowledge, no report can be found dealing with the characterization of cells expanded in a 3D fibrin-based environment. Russel et al. [25] compared the ability of PL and FBS to expand ADMSCs. They reported that both PL and FBS can be used to expand ADMSCs up to 20% medium supplementation, even if PL failed to obtain ADMSCs when SVF cells were plated directly in a medium supplemented with this platelet concentrate. The authors concluded that canine PL is inferior to FBS for the propagation of ADMSCs. Our results are partially at odds. Although we did not plated SVF directly in PL supplemented medium in 2D culture, we could efficiently expand SVF cells in a 3D fibrin-based, matrix environment. Furthermore, the use of 10 to 20% of PL for the expansion of ADMSCs on 2D plastic surface provided a stimulus to cell proliferation comparable to that provided by the FBS supplementation. Finally, a 3D fibrin-based environment deprived of xenobiotic supplementation was shown to be efficient in ADMSCs expansion.

A further aspect to be explored is the precise role of the 3D fibrin scaffold as well as that of the blood-derived product we have evaluated on the ADMSCs and SVF proliferation. The present research does not provide any conclusive data that support one of the different possibilities that could explain a higher replicative rate inside the 3D scaffold. We could hypothesize that the 3D environment itself provides a larger space for cell growth, and/or the molecular components of serum and platelet concentrates could represent a stimulus for cell replication. As a matter of fact, both 3D fibrin matrix [28] and platelet concentrates [23,24,25,26] represent complex biological cell growth-supplement able to sustain cell survival and replication. Recently, Kakudo et al. analyzed the role of PL on human adipose-derived MSCs, demonstrating that the already known proliferative effect is possibly caused by the activation of multiple signaling pathways, as observed for FBS [53]. Furthermore, in our experimental conditions the role of 3D fibrin scaffold could mimic a “natural extracellular matrix”, in terms of three-dimensional structure and composition. These characteristics, in combination with a wider space available for cell growth, are key features of fibrin meshwork [28]. This hypothesis is supported by the fact that cell doubling time inside the fibrin matrix is lower - thus ensuring a higher rate of cell growth - at 48 and 72 h, with respect to plastic surface where cell expansion is still active. From 72 to 144 h time-points, cell growth on plastic is compromised while in the 3D environment the same DT is maintained, providing evidence that further space is available for cell growth. Since no difference in cell replication was observed for 3D scaffolds prepared by using either PPP or PRP, we can argue that the whole characteristics of fibrin network, i.e., its chemical, biological, and mechanical properties, as well as the large space available play a complex pivotal role. Further studies are needed to clarify these issues and to explain the molecular patterns involved.

Our work aimed to assess if canine plasma, PRP, and PL can be used to set-up xenobiotic-free culture systems of ADMSCs. Our results support the hypothesis that canine species-specific substitutes to FBS can be used to prepare a large number of ADMSCs suitable for clinical application. Furthermore, it is possible to hypothesize the use of an autologous plasma-derived supplement to reduce the risk of transmission of pathogens to the patient, associated with the use of xeno- or allogeneic supplements.

## 5. Conclusions

Our results suggest that allogeneic and autologous blood-derived products can be used as a substitute for FBS in the expansion of canine ADMSCs. Furthermore, PRP, PL and dog serum can be used to generate a 3D matrix that allows a rapid expansion of ADMSCs and SVF obtained from the enzymatic digestion of adipose tissue. The use of homospecific blood derivatives and a 3D environment for ADMSCs and SVF cultures offers several advantages. First of all, the use of homospecific products reduces the potential risk of adverse immune reactions in patients, associated with the use of FBS. The use of autologous blood derivatives also minimizes the risk of disease transmission since the supplement for cell growth is derived from the same animal. Furthermore, homospecific blood derivatives, when used for the preparation of a three-dimensional growth environment, improve the timing of cell preparation and ensure the possibility of obtaining a large number of cells in a limited time compared to cultures on plastic. A further advantage of the use of a 3D environment prepared with an autologous serum is that a limited amount of blood sample is needed for cell amplification. Finally, the use of autologous fibrin-based matrices suggests the clinical feasibility of point-of-care applications based on their combination with ADMSCs or SVF, showing the possibility of interesting therapeutic perspectives.

## Figures and Tables

**Table 1:** Average cell number, doubling time and cell doubling number ±SD obtained at different time points (48, 72, 144, 216 h) from ADMSCs cultured on 2D plastic surface or 3D fibrin-based gel prepared from PPP (n = 5). Cells were seeded at a density of 6000 cells/cm^2^ in 3.5 cm Petri dishes.

		48 h	72 h	144 h	216 h
**Average Cell Number ± SD**	Plastic	153,378 ± 39,898	293,887 ± 110,270	516,917 ± 201,761	887,667 ± 406,264
	3D matrix	255,500 ± 82,526	576,136 ± 280,021	3,550,000 ± 546,493	3,095,000 ± 877,605
**Average Doubling Time ± SD**	Plastic	37.7 ± 11.1	34.3 ± 9.8	48.5 ± 9.2	57.4 ± 7.7
	3D matrix	24.4 ± 6.2	23.6 ± 4.9	24.3 ± 0.9	38.3 ± 3.7
**Average Cell Doubling Number ± SD**	Plastic	1.4 ± 0.4	2.2 ± 0.6	3.1 ± 0.6	3.8 ± 0.6
	3D matrix	2.1 ± 0.5	3.2 ± 0.7	5.9 ± 0.2	5.7 ± 0.5

**Table 2:** Average cell number, doubling time and cell doubling number obtained at different time points (72, 144 h) from ADMSCs cultured inside a 3D fibrin-based matrix prepared from PRP or PPP (n = 3).

		72 h	144 h
**Average Cell Number ± SD**	PRP 3D matrix	431,889 ± 120,870	2,707,556 ± 1,630,518
	PPP 3D matrix	406,889 ± 141,420	2,623,334 ± 1,470,181
**Average Doubling Time ± SD**	PRP 3D matrix	26.2 ± 4.6	27.8 ± 5.1
	PPP 3D matrix	27.7 ± 6.5	27.9 ± 5.2
**Average Cell Doubling Number ± SD**	PRP 3D matrix	2.8 ± 0.5	5.3 ± 0.9
	PPP 3D matrix	2.7 ± 0.6	5.9 ± 0.9

**Table 3:** The average number of cells and cell number fold increase obtained from adipose tissue derived SVF cells expanded on plastic surface or inside a 3D matrix prepared from PPP. The cells were counted when plastic cultures reached about 90% of confluency (average 192 h) (n = 3).

		192 h
**Average Cell Number ± SD**	Plastic	1,161,455 ± 892,737
	3D matrix	2,272,750 ± 974,489
**Fold Increase**		2.49 ± 1.68

**Table 4:** Phenotypic characterization of ADMSCs and adipose tissue-derived SVF by RT-PCR.

		ADMSCs—Plastic Surface	ADMSCs—3D Matrix	SVF—3D Matrix
**MSCs Markers**	CD13	+	+	+
	CD29	+	+	+
	CD31	+	+	+
	CD34	-	+	+
	CD44	+	+	+
	CD45	-	-	+/--
	CD73	+	+	+
	CD90	+	+	+
	CD105	+	+	+
**Inflammation Modulators**	TSG-6	+	+	+
	IRAP	+	+	+
	SDF-1	-	+	+
	STC-1	-	+	+

## Supplementary Materials

The following are available online at https://www.mdpi.com/2073-4409/9/12/2578/s1, Video S1: ADMSCs cultured inside the 3D fibrin-based matrix. Title: ADMSCs cultured inside the 3D fibrin-based matrix, at seeding and after 144 h.

## Appendix A

**Table A1:** Markers used for the evaluation of gene expression in canine ADMSCs and SVF cells by RT-PCR.

MSCs Markers	Accession n.	Primers Sequences	Length of Amplicons
CD13	NM_001146034.1	Fw: GGTCCTTACCATCACCTGGC Rv: CCTAAGGCCATCCATCGTCC	335 bp
CD29	XM_005616949.1	Fw: AGGATGTTGACGACTGCTGG Rv: ACTTTGCATTCAGTGTTGTGC	356 bp
CD31	XM_538830.1	Fw: GCCCGAAGTTCACTCTCAAG Rv: CACTCCTTTGACCCACACCT	410 bp
CD34	NM_001003341.1	Fw: GAGATCACCCTAACGCCTGG Rv: GGCTCCTTCTCACACAGGAC	383 bp
CD44	NM_001197022.1	Fw: CCCCATTACCAAAGACCACGA Rv: TTCTCGAGGTTCCGTGTCTC	408 bp
CD45	XM_005622282.1	Fw: TGTTTCCAGTTCTGTTTCCCCA Rv: TCAGGTACAAAGCCTTCCCA	432 bp
CD73	XM_532221.4	Fw: GATGGGAAAGGCAAGAAGGCT Rv: TTCCTGGCATCTTGCTACGG	317 bp
CD90	NM_001287129.1	Fw: AAGCCAGGATTGGGGATGTG Rv: TGTGGCAGAGAAAGCCTTCCCA	285 bp
CD105	XM_005625330.2	Fw: GGTTCACTGCATCAACATGG Rv: AAGCTGAAGCGCACATCACC	279 bp

**Table A2:** Primers used to verify the expression of “inflammation modulators” in canine ADMSCs and SVF cells.

Inflammation Modulators	Accession n.	Primers Sequences	Length of Amplicons
IRAP	NM_001003096	Fw: CATGCTAGAACCATGGAAACCTGCAGG Rv: CCGGCTCGAGTTATTCCTTCTGGAAG	550 bp
SDF-1	NM_001308461.1	Fw: GCCGATTCTTCGAGAGCCAC Rv: TCTGCCATACGCTGTTAGCTT	240 bp
TSG-6	XM_533354.3	Fw: ATGATCATCTTTATTTCCTTATTTGTCTTG Rv: TTATAAATGGCTAAATCTTCCAGCTAAAAAG	833 bp
STC-1	XM_543238.5	Fw: TGATCAGTGCTTCTGCAACC Rv: TCACAGTCCAGTAGGCTTCG	466 bp

## References

[1] Tsang E.J., Zhu M., Ashjian P., De Ugarte D.A., Huang J.I., Mizuno H., Alfonso Z.C., Fraser J.K., Benhaim P., Hedrick M.H. (2002). Human Adipose Tissue Is a Source of Multipotent Stem Cells. Mol. Biol. Cell.

[2] Gimble J.M., Guilak F., A Bunnell B. (2010). Clinical and preclinical translation of cell-based therapies using adipose tissue-derived cells. Stem Cell Res. Ther..

[3] Si Z., Wang X., Sun C., Kang Y., Xu J., Wang X., Hui Y. (2019). Adipose-derived stem cells: Sources, potency, and implications for regenerative therapies. Biomed. Pharmacother..

[4] Takemitsu H., Zhao D., Yamamoto I., Harada Y., Michishita M., Arai T. (2012). Comparison of bone marrow and adipose tissue-derived canine mesenchymal stem cells. BMC Vet. Res..

[5] Strioga M., Viswanathan S., Darinskas A., Slaby O., Michalek J. (2012). Same or Not the Same? Comparison of Adipose Tissue-Derived Versus Bone Marrow-Derived Mesenchymal Stem and Stromal Cells. Stem Cells Dev..

[6] Shariatzadeh M., Song J., Wilson S.L. (2019). The efficacy of different sources of mesenchymal stem cells for the treatment of knee osteoarthritis. Cell Tissue Res..

[7] Musiał-Wysocka A., Kot M., Majka M. (2019). The Pros and Cons of Mesenchymal Stem Cell-Based Therapies. Cell Transplant..

[8] Hoffman A.M., Dow S.W. (2016). Concise Review: Stem Cell Trials Using Companion Animal Disease Models. Stem Cells.

[9] De Bakker E., Van Ryssen B., De Schauwer C., Meyer E. (2013). Canine mesenchymal stem cells: State of the art, perspectives as therapy for dogs and as a model for man. Vet. Q..

[10] Arzi B., Clark K.C., Sundaram A., Spriet M., Verstraete F.J., Walker N.J., Loscar M.R., Fazel N., Murphy W.J., Vapniarsky N. (2017). Therapeutic Efficacy of Fresh, Allogeneic Mesenchymal Stem Cells for Severe Refractory Feline Chronic Gingivostomatitis. Stem Cells Transl. Med..

[11] Gardin C., Ferroni L., Bellin G., Rubini G., Barosio S., Zavan B. (2018). Therapeutic Potential of Autologous Adipose-Derived Stem Cells for the Treatment of Liver Disease. Int. J. Mol. Sci..

[12] Dias I.E., Pinto P.O., Barros L.C., Viegas C.A., Dias I.R., Carvalho P.P., Dias I.E., Pinto P.O., Barros L.C., Viegas C.A. (2019). Mesenchymal stem cells therapy in companion animals: Useful for immune-mediated diseases?. BMC Vet. Res..

[13] Thomson A.L., Berent A.C., Weisse C., Langston C.E. (2019). Intra-arterial renal infusion of autologous mesenchymal stem cells for treatment of chronic kidney disease in cats: Phase I clinical trial. J. Vet. Intern. Med..

[14] Zeira O., Asiag N., Aralla M., Ghezzi E., Pettinari L., Martinelli L., Zahirpour D., Dumas M.P., Lupi D., Scaccia S. (2015). Adult autologous mesenchymal stem cells for the treatment of suspected non-infectious inflammatory diseases of the canine central nervous system: Safety, feasibility and preliminary clinical findings. J. Neuroinflamm..

[15] Bora P., Majumdar A.S. (2017). Adipose tissue-derived stromal vascular fraction in regenerative medicine: A brief review on biology and translation. Stem Cell Res. Ther..

[16] Laffey J., Hayes M. (2011). Faculty Opinions recommendation of Minimal criteria for defining multipotent mesenchymal stromal cells. The International Society for Cellular Therapy position statement. Faculty Opinions—Post-Publication Peer Review of the Biomedical Literature.

[17] Pak J., Lee J.H., Park K.S., Park M., Kang L.-W., Lee S.H. (2017). Current use of autologous adipose tissue-derived stromal vascular fraction cells for orthopedic applications. J. Biomed. Sci..

[18] Marx C., Silveira M.D., Selbach I., Da Silva A.S., Braga L.M.G.D.M., Camassola M., Nardi N.B. (2014). Acupoint Injection of Autologous Stromal Vascular Fraction and Allogeneic Adipose-Derived Stem Cells to Treat Hip Dysplasia in Dogs. Stem Cells Int..

[19] Von Bonin M., Stölzel F., Goedecke A., Richter K., Wuschek N., Hölig K., Platzbecker U., Illmer T., Schaich M., Schetelig J. (2008). Treatment of refractory acute GVHD with third-party MSC expanded in platelet lysate-containing medium. Bone Marrow Transplant..

[20] Allogenic Mesenchymal Stem Cell-Based Products for Veterinary Use: Specific Questions on Tumorigenicity. https://www.ema.europa.eu/en/allogenic-mesenchymal-stem-cell-based-products-veterinary-use-specific-questions-tumorigenicity.

[21] Johnson V., Webb T., Norman A., Coy J., Kurihara J., Regan D., Dow S. (2017). Activated Mesenchymal Stem Cells Interact with Antibiotics and Host Innate Immune Responses to Control Chronic Bacterial Infections. Sci. Rep..

[22] Quimby J.M., Webb T.L., Habenicht L.M., Dow S.W. (2013). Safety and efficacy of intravenous infusion of allogeneic cryopreserved mesenchymal stem cells for treatment of chronic kidney disease in cats: Results of three sequential pilot studies. Stem Cell Res. Ther..

[23] Kocaoemer A., Kern S., Klüter H., Bieback K. (2007). Human AB Serum and Thrombin-Activated Platelet-Rich Plasma Are Suitable Alternatives to Fetal Calf Serum for the Expansion of Mesenchymal Stem Cells from Adipose Tissue. Stem Cells.

[24] Naskou M.C., Sumner S.M., Chocallo A., Kemelmakher H., Thoresen M., Copland I., Galipeau J., Peroni J.F. (2018). Platelet lysate as a novel serum-free media supplement for the culture of equine bone marrow-derived mesenchymal stem cells. Stem Cell Res. Ther..

[25] Russell K.A., Gibson T.W.G., Chong A., Co C., Koch T.G. (2015). Canine Platelet Lysate Is Inferior to Fetal Bovine Serum for the Isolation and Propagation of Canine Adipose Tissue- and Bone Marrow-Derived Mesenchymal Stromal Cells. PLoS ONE.

[26] Chapman H.-S., Gale A.L., Dodson M.E., Linardi R.L., Ortved K.F. (2020). Autologous Platelet Lysate Does Not Enhance Chondrogenic Differentiation of Equine Bone Marrow-Derived Mesenchymal Stromal Cells Despite Increased TGF-β1 Concentration. Stem Cells Dev..

[27] Park C.H., Woo K.M. (2018). Fibrin-Based Biomaterial Applications in Tissue Engineering and Regenerative Medicine. Adv. Exp. Med. Biol..

[28] De La Puente P., Ludeña D. (2014). Cell culture in autologous fibrin scaffolds for applications in tissue engineering. Exp. Cell Res..

[29] Zubin E., Conti V., Leonardi F., Zanichelli S., Ramoni R., Grolli S. (2015). Regenerative therapy for the management of a large skin wound in a dog. Clin. Case Rep..

[30] Vidal M.A., Bs G.E.K., Johnson J.R., Lopez M.J., Moore R.M., Gimble J.M. (2006). Cell Growth Characteristics and Differentiation Frequency of Adherent Equine Bone Marrow—Derived Mesenchymal Stromal Cells: Adipogenic and Osteogenic Capacity. Vet. Surg..

[31] Doubling Time Computing. http://www.doubling-time.com/compute.php.

[32] Ivanovska A., Grolli S., Borghetti P., Ravanetti F., Conti V., De Angelis E., Macchi F., Ramoni R., Martelli P., Gazza F. (2017). Immunophenotypical characterization of canine mesenchymal stem cells from perivisceral and subcutaneous adipose tissue by a species-specific panel of antibodies. Res. Vet. Sci..

[33] De Schauwer C., Van De Walle G.R., Van Soom A., Meyer E. (2013). Mesenchymal stem cell therapy in horses: Useful beyond orthopedic injuries?. Vet. Q..

[34] Barrachina L., Romero A., Zaragoza P., Rodellar C., Vázquez F.J. (2018). Practical considerations for clinical use of mesenchymal stem cells: From the laboratory to the horse. Vet. J..

[35] Tanavde V., Vaz C., Rao M.S., Vemuri M.C., Pochampally R. (2015). Research using Mesenchymal Stem/Stromal Cells: Quality metric towards developing a reference material. Cytotherapy.

[36] Pérez-Merino E., Usoncasaus J.M., Zaragoza-Bayle C., Duque-Carrasco J., Mariñas-Pardo L., Hermida-Prieto M., Barrera-Chacón R., Gualtieri M. (2015). Safety and efficacy of allogeneic adipose tissue-derived mesenchymal stem cells for treatment of dogs with inflammatory bowel disease: Clinical and laboratory outcomes. Vet. J..

[37] Martin-Rufino J.D., Sánchez F.S.L., Redondo A.M., Villarón E.M., Rueda R., Fernandez-Samos R., Sanchez-Guijo F. (2018). Sequential intravenous allogeneic mesenchymal stromal cells as a potential treatment for thromboangiitis obliterans (Buerger’s disease). Stem Cell Res. Ther..

[38] Yao Y., Dong Z., Liao Y., Zhang P., Ma J., Gao J., Lu F. (2017). Adipose Extracellular Matrix/Stromal Vascular Fraction Gel. Plast. Reconstr. Surg..

[39] Sullivan M.O., Gordon-Evans W.J., Fredericks L.P., Kiefer K., Conzemius M.G., Griffon D.J. (2016). Comparison of Mesenchymal Stem Cell Surface Markers from Bone Marrow Aspirates and Adipose Stromal Vascular Fraction Sites. Front. Vet. Sci..

[40] Becherucci V., Piccini L., Casamassima S., Bisin S., Gori V., Gentile F., Ceccantini R., De Rienzo E., Bindi B., Pavan P. (2018). Human platelet lysate in mesenchymal stromal cell expansion according to a GMP grade protocol: A cell factory experience. Stem Cell Res. Ther..

[41] Li Y., Meng H., Liu Y., Lee B.P. (2015). Fibrin Gel as an Injectable Biodegradable Scaffold and Cell Carrier for Tissue Engineering. Sci. World J..

[42] Reinke J., Sorg H. (2012). Wound Repair and Regeneration. Eur. Surg. Res..

[43] Dykstra J.A., Facile T., Patrick R.J., Francis K.R., Milanovich S., Weimer J.M., Kota D.J. (2017). Concise Review: Fat and Furious: Harnessing the Full Potential of Adipose-Derived Stromal Vascular Fraction. Stem Cells Transl. Med..

[44] Tambella A.M., Attili A.R., Dupré G., Cantalamessa A., Martin S., Cuteri V., Marcazzan S., Del Fabbro M. (2018). Platelet-rich plasma to treat experimentally-induced skin wounds in animals: A systematic review and meta-analysis. PLoS ONE.

[45] Tsai H.-C., Chang G.R.-L., Fan H.-C., Ou-Yang H., Huang L.-C., Wu S.-C., Chen C.-M. (2019). A mini-pig model for evaluating the efficacy of autologous platelet patches on induced acute full thickness wound healing. BMC Vet. Res..

[46] Yin S., Yang X., Bi H., Zhao Z. (2020). Combined Use of Autologous Stromal Vascular Fraction Cells and Platelet-Rich Plasma for Chronic Ulceration of the Diabetic Lower Limb Improves Wound Healing. Int. J. Low. Extrem. Wounds.

[47] Angelone M., Conti V., Biacca C., Battaglia B., Pecorari L., Piana F., Gnudi G., Leonardi F., Ramoni R., Basini G. (2017). The Contribution of Adipose Tissue-Derived Mesenchymal Stem Cells and Platelet-Rich Plasma to the Treatment of Chronic Equine Laminitis: A Proof of Concept. Int. J. Mol. Sci..

[48] Bartosh T.J., Ylostalo J.H. (2019). Efficacy of 3D Culture Priming is Maintained in Human Mesenchymal Stem Cells after Extensive Expansion of the Cells. Cells.

[49] Harrell C.R., Markovic B.S., Fellabaum C., Arsenijevic N., Djonov V., Volarevic V. (2020). The role of Interleukin 1 receptor antagonist in mesenchymal stem cell-based tissue repair and regeneration. BioFactors.

[50] Luo Q., Zhang B., Kuang D., Song G. (2016). Role of Stromal-derived Factor-1 in Mesenchymal Stem Cell Paracrine-mediated Tissue Repair. Curr. Stem Cell Res. Ther..

[51] Bartosh T.J., Ylostalo J.H., Bazhanov N., Kuhlman J., Prockop D.J. (2013). Dynamic compaction of human mesenchymal stem/precursor cells into spheres self-activates caspase-dependent IL1 signaling to enhance secretion of modulators of inflammation and immunity (PGE2, TSG6, and STC1). Stem Cells.

[52] Shanbhag S., Stavropoulos A., Suliman S., Hervig T., Mustafa K. (2017). Efficacy of Humanized Mesenchymal Stem Cell Cultures for Bone Tissue Engineering: A Systematic Review with a Focus on Platelet Derivatives. Tissue Eng. Part B Rev..

[53] Kakudo N., Morimoto N., Ma Y., Kusumoto K. (2019). Differences between the Proliferative Effects of Human Platelet Lysate and Fetal Bovine Serum on Human Adipose-Derived Stem Cells. Cells.

